# Supplementary material for: Addressing the polypharmacy challenge in older people with multimorbidity (APOLLO-MM): study protocol for an in-depth ethnographic case study in primary care
Source: BMJ Open. 2019 Aug 22;9(8):e031601. doi: 10.1136/bmjopen-2019-031601 (PMC6707689; doi:10.1136/bmjopen-2019-031601)
Supplement: Author's manuscript [file bmjopen-2019-031601.draft_revisions.pdf]

BMJ Open

BMJ Open is committed to open peer review. As part of this commitment we make the peer review history of every article we publish publicly available.

When an article is published we post the peer reviewers' comments and the authors' responses online. We also post the versions of the paper that were used during peer review. These are the versions that the peer review comments apply to.

The versions of the paper that follow are the versions that were submitted during the peer review process. They are not the versions of record or the final published versions. They should not be cited or distributed as the published version of this manuscript.

BMJ Open is an open access journal and the full, final, typeset and author-corrected version of record of the manuscript is available on our site with no access controls, subscription charges or pay-per-view fees (http://bmjopen.bmj.com).

If you have any questions on BMJ Open's open peer review process please email info.bmjopen@bmj.com

BMJ Open

Addressing the Polypharmacy Challenge in Older People with Multimorbidity (APOLLO-MM): an in-depth ethnographic case study

Journal:	*BMJ Open*
Manuscript ID	bmjopen-2019-031601
Article Type:	Protocol
Date Submitted by the Author:	10-May-2019

Complete List of Authors:	Swinglehurst, Deborah; Queen Mary University of London Faculty of Medicine and Dentistry, Centre for Primary Care and Public Health<br>Fudge, Nina ; Queen Mary University of London Faculty of Medicine and Dentistry, Centre for Primary Care and Public Health

Keywords:	polypharmacy, multimorbidity, ethnography, PRIMARY CARE

SCHOLARONE™
Manuscripts

TITLE: Addressing the Polypharmacy Challenge in Older People with Multimorbidity (APOLLO-MM): an in-depth ethnographic case study

Authors: Deborah Swinglehurst*, Nina Fudge

*Corresponding author

CONTACT DETAILS

Professor Deborah Swinglehurst

Centre for Primary Care and Public Health

Blizard Institute

Barts and The London School of Medicine and Dentistry

Queen Mary University of London

Yvonne Carter Building

58 Turner Street

London E1 2AB

UK

Telephone: +44 (0)20 7882 7204

E mail: d.swinglehurst@qmul.ac.uk

ORCID ID: https://orcid.org/0000-0003-1261-9268

Dr Nina Fudge (same institutional address as DS)

Telephone: +44 (0)20 7882 2528

E mail: n.fudge@qmul.ac.uk

ORCID ID: http://orcid.org/0000-0002-7161-4355

WORD COUNT: 3990

For peer review only - http://bmjopen.bmj.com/site/about/guidelines.xhtml

ABSTRACT

Introduction

Polypharmacy is on the rise. It is burdensome for patients and is a common source of error and adverse drug reactions, especially amongst older adults. Health policy advises clinicians to practice *medicines optimisation* – a person-centred approach to safe, effective medicines use. There has been little research exploring older patients' perspectives and priorities around medicines-taking, nor their actual practices of fitting medicines into their daily lives and how these are shaped by the wider context of health care.

Methods and Analysis

We will conduct an in-depth multisite ethnographic case study. The study is based in 7 clinical sites (3 general practices; 4 community pharmacies) and includes longitudinal ethnographic follow-up of older adults, organisational ethnography and participatory methods. Main data sources include: field notes of observations in the home and clinical settings; interviews with patients and professionals; cultural probe activities; video-recordings of clinical consultations and inter-professional talk; documents. Our analysis will illuminate: the everyday practices of polypharmacy from a range of lay and professional perspectives; the institutional contexts within which these practices play out; and the sense-making work that sustains - or challenges - these practices. Our research will adopt a 'practice theory' lens, drawing on the sociology of organisational routines, and other relevant social theory guided by ongoing iterative data analysis.

Ethics Approval

The study has HRA approval and received a favourable ethical opinion from the Leeds West Research Ethics Committee (IRAS project ID: 205517; REC reference 16/YH/0462).

Dissemination

Aside from academic outputs, our findings will inform the development of recommendations for practice and policy including an interactive e-learning resource. We will also work with service users to co-design patient/public engagement resources.

For peer review only - http://bmjopen.bmj.com/site/about/guidelines.xhtml

Keywords: polypharmacy; multimorbidity; ethnography; primary care

ARTICLE SUMMARY: STRENGTHS AND LIMITATIONS OF THIS STUDY

- Gathers rich qualitative data across community settings (the home; general practice; pharmacy) with a focus on how practices unfold on the ground and how different actors make sense of polypharmacy
- Brings together analysis of patients' lived experience with analysis of organisational routines and professional practices
- Uses innovative qualitative methodologies which generate more sophisticated insights than is possible with conventional approaches
- Prioritises depth of analysis over breadth, increasing richness of understanding but limiting generalisability of findings.

INTRODUCTION

Polypharmacy, usually defined as the co-prescription of five or more medications (1) is increasing. One Scottish study showed that about 20% of adults are dispensed five or more drugs (this doubled between 1995 and 2010) and about 6% of adults are dispensed 10 or more drugs (this tripled over the same time period) (2). Polypharmacy is burdensome for patients, and is a common source of error. Research in primary care suggests there are errors in 5% of prescription items (3) with polypharmacy a specific risk factor for error.

Polypharmacy arises in part because of an ageing population of people with multiple medical problems. Polypharmacy may sometimes be appropriate, but there is growing concern that it is one example of 'medical overuse' causing unnecessary burden and iatrogenic harm to patients (falls, adverse drug reactions, hospital admissions for example) and costly waste to health systems. Up to half of medicines prescribed for long term conditions are unused (4). This is often conceptualised as a problem of poor adherence (5-7) or as a failure of shared decision-making in the consultation (8, 9). The problem is assumed to be located within individuals' behaviours, with relatively little attention paid to the relationship between individuals and the wider social, organisational and institutional context within which their medicines practices play out. Furthermore, although polypharmacy is often attributed to the ageing population (65% of people aged 65 – 84, and over 80% of people aged 85+ experience multimorbidity (10)), this is just one of many potentially relevant factors.

Evidence-based guidance, typically arising from randomised controlled trials (RCTs) is organised around a 'single disease' model which may compound rather than alleviate polypharmacy. Interpreting evidence from RCTs in the context of an older person with numerous medical problems is difficult. One critic has described evidence based medicine as a perverse 'monument to bias', built on its systematic exclusion of complex patients with multimorbidity from the trials that inform it (11).

For peer review only - http://bmjopen.bmj.com/site/about/guidelines.xhtml

Arguably polypharmacy in the context of multimorbidity is rarely, if ever, evidence-based, even when a clear ‘evidence-based’ argument can be made for individual items (12).

Achieving *medicines optimisation* – a person-centred approach to safe, effective medicines use (13-15) - is fraught with difficulty in an institutional context which is largely organised around a ‘single disease’ model and is increasingly concerned with the application of abstract, generalisable rules. This is further complicated by increasing emphasis on what has been called ‘risk factorology’ and the quest to eliminate risk of disease altogether (16).The societal context is increasingly one in which “every responsible and rational citizen is expected to actively seek out and eliminate all possible risks to their future health and to consume medical technologies in order to achieve this aim” (17). In recent years the UK Quality and Outcomes Framework and other similar pay-for-performance initiatives embrace (and arguably constitute) these aspirations to manage disease and eliminate risk and uncertainty. A recent study which examined temporal trends between 2000 and 2015 in the use of tests and investigations in UK primary care found that age- and sex-adjusted use of tests and investigations increased by a staggering 8.5% *annually* (18). Although prevalence studies do not offer insight into the drivers of change, it seems reasonable to conclude that the parallel and dramatic rise in investigations and polypharmacy over a similar time period may be related phenomena. Privileging priorities for risk reduction in one area may contribute paradoxically, and at great cost, to new drug-related risks – risks which increase with increasing age and with increasing numbers of medications (19). ‘Problematic’ polypharmacy (15) arises insidiously, despite (and sometimes even because of) clinicians and patients striving to achieve best practice and acting with the very best of intentions. The elusive boundary between ‘just enough medicine’ and ‘too much medicine’ is shifting, unstable, inherently contingent and sits firmly within the realm of clinical judgement in the context of incomplete information and uncertainty. Sinnott has referred to the work of negotiating this boundary as a process of ‘satisficing’ (20). Doctors report settling for management that is satisfactory and sufficient given the particular circumstances of a patient - a combination of relaxing nationally recommended targets, compromise, hunch, guesswork and maintaining the status quo when patients appear stable (20).

Polypharmacy represents a complex challenge for clinicians and health systems. It is a ‘wicked problem’ (21). It arises at the interface of patients, clinicians, diseases (and risk thereof) and involves a tangle of biological, cultural, technological, economic and socio-political dimensions. One of the features of wicked problems is their resistance to adequate definition. A recent systematic review identified 138 definitions of polypharmacy in the academic literature, most of them numerical definitions only (1). But simple numerical definitions conceal much of the complexity that the polypharmacy concept evokes (19). Rittel states that “the formulation of a wicked problem *is* the problem”; wicked problems are ill-defined, not amenable to a quick-fix ‘solutions’ but at best ‘*resolved, or rather re-solved, over and over again*’. Wicked problems coalesce around important moral issues whereby there are acknowledged discrepancies between the state of affairs as it *is* and the state as it *ought* to be (21). Ethnographic approaches are especially good at investigating complex issues such as polypharmacy and shedding light on ill-defined but troubling phenomena (22).

For peer review only - http://bmjopen.bmj.com/site/about/guidelines.xhtml

Aim

The aim of the APOLLO-MM project is to improve patient care by producing 'practice-based' evidence (23) to inform medicines optimisation. We will do this by:

- Examining patients' experiences of polypharmacy to discern elements of high quality 'medicines work' and those elements that might lead to harmful, wasteful or unnecessary polypharmacy
- Improving understanding of how polypharmacy is sustained and/or challenged within and between lay and professional networks
- Analysing consultations that include talk about medicines (including but not limited to formal 'medication review') to understand how medicine optimisation is negotiated in practice
- Using participatory methods to elicit professional dialogue around polypharmacy and offer opportunities to discern the elements of good practice in medicines optimisation
- Working with patients and professionals in a process of co-design to develop e-learning materials, recommendations for practice and public/patient engagement resources

Research questions

1. What is the patient experience of polypharmacy in multimorbidity?
2. What does polypharmacy mean for patients and carers?
    a. How are patients' lives with multimorbidity shaped by practices of managing medicines?
    b. How is the managing of medicines shaped by contingencies of living with multimorbidity?
3. How do the practices of patients / carers / health professionals and wider systems of care support 'appropriate' polypharmacy or challenge 'inappropriate' polypharmacy and with what consequences for the quality and safety of care?
4. How can insights from longitudinal patient case studies inform improvements in professional practices, service delivery and public awareness of polypharmacy?

METHODS

Setting and Context

The study is a multicentre ethnographic case study involving seven clinical sites across two research clusters in England (one urban and one suburban cluster). The urban cluster comprises two NHS GP practices (Site A, Site C) and two independent community pharmacies located in an area of high socio-economic deprivation within a large city, serving a population of diverse ethnicity comprising approximately 40% Asian patients. The suburban cluster comprises one NHS GP practice (Site B) and two independent community pharmacies, located in a small, relatively prosperous city serving a population which is 85% white Caucasian. The research clusters represent contrasting demographics; the urban cluster serves patient populations within the lowest (Site C) and second lowest (Site A) decile of the English Index of

For peer review only

multiple deprivation score (24), and the suburban cluster serves a population within the least deprived decile.

The GP practices were selected following expressions of interest from over forty GP practices within the North Thames Clinical Research Network area, suggesting that the project resonates with the concerns of GPs on the ground.

We have recruited 24 patients, 14 in the urban cluster (7 each from site A and C) and 10 from our suburban cluster. The work of patient follow-up and organisational ethnography has begun. The GP practices and community pharmacies in the study share responsibility for prescribing and dispensing medicines to the study patients, allowing for multiple perspectives of polypharmacy. The relationship between the patients, GP practices and community pharmacies is shown in Figure 1.

[INSERT FIGURE 1 ABOUT HERE] Figure 1. Study sites and research clusters

Selection of organisational cases

Drawing on Stakes approach to organisational case study our selection of GP sites primarily reflects 1) *opportunity for learning*, rather than concerns about representativeness (25) and 2) diversity of geographical setting and population demographics. Our concern is to conduct an in-depth study of *particular* practices within these sites, adopting an interpretive approach informed by relevant social theory. We will create generalisable theoretical and conceptual abstractions (novel concepts to 'think with') rather than generalisable statistical abstractions. The ethnographic approach relies on the interest shown in the study by the potential participants, the willingness of staff at all levels of the organisation to allow meaningful access to the research team (e.g. shadowing, observations, formal and informal interviews; access to relevant documents) and enthusiasm for the participatory element of the study within the context of already heavy workloads (see *Study Design*). In addition we considered a range of pragmatic concerns relating to feasibility, location and likelihood of building sustainable working relationships for the duration of the study. Table 1 shows some of the characteristics of our recruited practices.

[INSERT TABLE 1 ABOUT HERE]

For peer review only

For peer review only - http://bmjopen.bmj.com/site/about/guidelines.xhtml

Table 1. Characteristics of recruited GP practices

	Site A	Site B	Site C
Cluster	Urban	Suburban	Urban
Patient population	c.11000	c.13000	c.14000
General Practitioners	5 partners; 5 salaried	4 partners; 6 salaried	8 partners; 3 salaried
On-site clinical pharmacist at recruitment	No	No	No
On-site clinical pharmacist for part of project duration	Yes	Yes	Yes
GP training practice	Yes	Yes	Yes
Deprivation*	2nd most deprived decile	Least deprived decile	Most deprived decile
Ethnic diversity (estimated proportion non-white ethnic groups)*	Over 50% (over 40% of practice population are Asian)	14%	Over 60% (over 40% of practice population are Asian)

**details from National General Practice Profiles produced by Public Health England Data Science et al. (REF)*

Sampling of patient participants

We have recruited patients aged 65 or over and prescribed 10 or more regular (repeat) items of medication, this being regarded a pragmatic marker of 'high risk polypharmacy' (15). Where patients were prescribed an item in more than one dose (e.g. warfarin), this was counted as a single item. Medical devices and hosiery were excluded, but medicines in all forms (e.g. oral, inhalers, injectables, topical creams etc) were included. We adopted a purposive sampling approach, aiming for a maximum diversity sample across a range of attributes: age (ranging from 65 – 94); gender (11 men; 13 women); living circumstances (16 live alone of whom 5 in sheltered housing; 8 with partners/family; 4 housebound); socioeconomic status (home ownership and previous occupation as proxy indicators); number of medication items (10 – 17). All patients have two or more comorbidities. 21 of 24 patients are white Caucasian. We have excluded patients unable to speak adequate English, which means that sampling in our urban cluster is not representative of the local population. We are addressing this limitation in another study (26). Patients lacking cognitive capacity to consent were eligible for inclusion but we were unable to recruit any patients who lacked capacity to consent. We are addressing this using a different recruitment strategy in a parallel study which focusses on patients with dementia (26). One patient (with dysphasia following a stroke) has a personal consultee.

Recruitment took place in general practice. GPs and nurses introduced the study to potential participants, issued participant information leaflets and sought verbal consent from patients to be contacted by a researcher (n=63). 36 patients agreed to a home visit to discuss the study in more detail and 24 consented to take part.

For peer review only - http://bmjopen.bmj.com/site/about/guidelines.xhtml

STUDY DESIGN, METHODOLOGY AND APPROACH TO ANALYSIS

Figure 2 shows the methods and data sources for the study, which are designed to elicit and explore patient experiences and practices; professional experiences and practices; professional dilemmas. These are described in detail in the following sections.

[INSERT FIGURE 2 ABOUT HERE] Figure 2: Methods and data sources

Patient experiences and practices

We will follow patient participants ethnographically over 18-24 months, observing them at home and accompanying them to selected health care interactions in which they anticipate talk about their medicines (e.g. at the GP surgery, pharmacy or hospital). We are conducting interviews with patients to elicit the story of their lives since they were first advised to take medicines, adapting Wengraf's Biographic-Narrative Interpretive Method (BNIM) (27). This approach utilises a single question to induce narrative (the SQUIN) and invites participants to speak uninterrupted until their story is complete. After a short break (10 – 15 minutes) the researcher presents a series of further questions based on 'cue phrases' (the patient's own words) in the order the patient introduced them into their opening narrative, to encourage further in-depth narratives (particular incident narratives or PINs). On a separate occasion we return to the patient's home to conduct an in-depth interview guided by a topic guide with a more explicit focus on medicines practices. We contact participants by telephone or home visit at approximately 4 – 8 weekly intervals to learn about their ongoing experiences and to identify occasions when we may accompany them to a health care interaction or video-record a consultation. Every time we meet our participants we take detailed ethnographic field notes, add to our reflexive journal and gather relevant documents (e.g. discharge summary or lists of medication). This data enables us to build detailed patient case studies as we follow their lived experience and engage them in informal 'event based' interviews exploring their care experiences.

A subset of patients are completing cultural probe activities as a way of depicting their lives. Cultural probes originated in the world of design as a way of tapping into peoples' creativity and offering research participants opportunity to engage in a research-related activity that is subjective, deliberately ambiguous, open to interpretation, invites imagination and stimulates conversation (28, 29). The probes consist of a set of activities and a range of materials (e.g. camera, pens, cards, booklets) with simple instructions which offer opportunity for improvisation. They encourage participants to think about their medicines and bodies in new ways and re-imagine possibilities for how they might interact with health professionals. The creative outputs are used to stimulate an informal discussion between researcher and participant. Participants may invite a family member or researcher to assist if they wish. Cultural probes have been used successfully to incorporate older people's ideas into the design of social communities (28), and to illuminate the daily living needs of older people to inform co-production of assistive living technologies (30, 31) although it is recognised that some people may be unable to engage with them for a range of physical, emotional and social reasons (30).

For peer review only - http://bmjopen.bmj.com/site/about/guidelines.xhtml

Professional experiences and practices

We will conduct ethnographic observations in three GP and four community pharmacy practices (see Figure 1). Our interest is in understanding how health professionals conduct and make sense of their day-to-day work of prescribing, dispensing, negotiating, organising medicines for patients affected by polypharmacy. We will observe key routines such as: preparation and organisation of multicompartment compliance aids ('dosette boxes'); repeat prescribing and repeat dispensing.

We will closely observe: individuals at work (shadowing, and asking participants to describe what they are doing as they do it to encourage the articulation of tacit knowledge); organisational routines and how they are constituted; the wider institutional context; documents relevant to the work we observe (e.g. prescriptions, guidelines). We will also interview key professional participants across our sites with a focus on accounts of their working practices. We will hone in on consultations between clinicians and our study patients to examine healthcare interactions in detail by video-recording consultations in which our participants anticipate 'talk about medicines'. The polypharmacy context is a very particular context for these interactions and we are interested to learn how polypharmacy is sustained, challenged, talked about and 'brought about' in these interactions.

Organisational routines are conceptualised as "repetitive recognisable patterns of interdependent actions by multiple actors" (32, 33) and can be regarded as sites of stability and as sites of potential for innovation and change (34). When participants engage in routines they enact tacit knowledge, and are involved in complex patterns of collaboration – involving people, artefacts, technologies - to get work done. Routines can be embodied, embraced, resisted, and enacted with varying degrees of creativity and improvisation, and varying attention to the particularities of local context (35). Pentland and Feldman have conceptualised routines as being comprised of:

- *ostensive routines* - the generalised 'rules' or organisational scripts that guide the routine, of which there may be many instantiations.
- *performative routines* - what people *actually do in practice* as they engage in the routine. Each instantiation of the routine is a unique performance.

Ostensive routines may be accessed through peoples' accounts of the work they do whilst observation of repeated rounds of a routine gives insight into the many versions of the performative routine and the relationship between the performative and the ostensive aspects of the routines. When people engage in routines they select from a repertoire of possible actions (enacting possibilities around which there are explicit or implicit constraints). Different people bring different knowledge, different expertise, different assumptions and different personal and organisational narratives to the enactment of the routine. An understanding of the patterns of possibility and constraints offers insights into wider organisation principles and values, and to an appreciation of complex organisational practices, such as how power circulates and how learning is done. The dynamic interplay between the ostensive and performative routines and the artefacts which are drawn upon to

For peer review only - http://bmjopen.bmj.com/site/about/guidelines.xhtml

support these routines can transform what has traditionally been regarded as mundane and ordinary organisational life (e.g. the processing of a repeat prescription) into opportunity for 'thick description' (36) of the nature of organisational life and the wider context within which work is being done (33, 37). Continuities and discontinuities in the relationship between ostensive and performative routines may become manifest as puzzles, conflicts, moments of perplexity or tensions.

Recent literature on organisational routines has pointed to the need to pay greater attention to the relational nature of 'self' and agency in the performance of routines and in the dynamic processes that address *how routines become collective accomplishments* with recognisable shared collective characteristics (i.e. how the 'ostensive' aspect of routines is constituted or brought about) (38, 39). One approach which offers promise is to locate the study of routines within emerging 'communication as constitutive of organization (CCO)' scholarship (40-43) wherein communication is itself framed as performance (through conversation and text dialectic). Routines are then framed as communicatively constituted performances, there is greater emphasis placed on performativity, embodiment, dialogic interrelationships, and interest in 'strings of associations' that link actors (human and non-human) as routines unfold (44). The nuance of communication gains ground - the emergence of organisational storylines, for example (45-47). This approach aligns with our interest in patient narratives and our orientation to 'talk about medicines' as co-constructed accomplishment within the consultation.

Video Reflexive Ethnography (VRE)

Video-reflexive ethnography is an innovative approach to quality and safety research in health care (48-50) which encourages groups of professionals to discuss and reflect on their working practice. In this study, selected video-recorded footage of 'medicines talk' in our study patients' consultations will be shown to small groups of professionals across our research sites. VRE offers an opportunity for participants to negotiate shared meanings in their practice, engage with the complexity of their everyday work, and make visible - in new ways - aspects of their practice which have become habituated, taken-for- granted, and 'invisible' (50). It is a participatory approach in which researchers and practitioners work together to illuminate this complexity and foster dialogue that might not otherwise occur (51). The footage acts as a catalyst, making implicit knowledge explicit through a process of reflection-on-action which renders the work (in this case the practical work of 'medicines optimisation') understandable in new ways and open to new ways of re-imagining practice (52). Importantly the discussion may go beyond what is visible in the footage, the footage acting as a prompt to inter-professional exchange and peer-led discussion of professional concerns, dilemmas and opportunities. There is opportunity for 'exnovation' or innovation from *within* practice (49) and the cultivation of meta-discourses of practice (51) which can reframe everyday practices (for example the 'medication review') by drawing out the systemic implications of what specific people know, do and say. By video-recording the discussion this meta-discourse can be teased out and worked with across VRE sessions, in ways which

For peer review only - http://bmjopen.bmj.com/site/about/guidelines.xhtml

enable practitioners to iteratively reframe their work and negotiate new possibilities for action. Importantly the collaborative process of co-constructing this meta-discourse in the VRE context may itself be a rehearsal space for new ways of relating and collaborating in the very doing of the work that is under scrutiny.

Participatory co-design

The findings from the research will feed into a process of participatory co-design, working in collaboration with both patients and professionals to design e-learning materials for professional education and patient engagement resources for patients. In this work we will adapt processes of experience-based co-design (53, 54) and 'future groups' (55), the latter being a reinterpretation of focus group methods which uses ideation tools and encourages speculation to imagine alternative approaches to care. We will collaborate with a design researcher to develop our research materials and findings to create bespoke ideation tools and props to facilitate imaginative participation and move towards exploring future alternatives to care prompted by the stories, data and findings from the earlier research.

Integrating analysis across diverse datasets

The project is situated within a broad 'practice theory' orientation and adopts a social constructionist orientation. Practice theory encourages an understanding of work as an accomplishment – and fosters a curiosity about what is accomplished by whom and how. The organisation (e.g. the GP practice or pharmacy) is understood as both the *site* and the *result* of work activities and the role of the interpretive researcher is to 'zoom in' on the details of particular practices and to 'zoom out' to discern the relationships, and connections between people, routines, artefacts, spaces and technologies for example (56, 57).

This detailed ethnography will extend our previous research on repeat prescribing in general practice settings in four ways (37). Firstly the institutional context has changed (one example being the implementation of the Electronic Prescribing Service, another being the incorporation of clinical pharmacists within some GP settings). Secondly, our research extends beyond general practice into community pharmacy settings and into peoples' homes and opens up a wider range of vantage points from which to observe the phenomenon. We are focussing our observations around the phenomenon of polypharmacy, recognising that polypharmacy emerges out of an arrangement of practices and is not a discrete entity that can be easily observed without appreciation of a wider social context. Finally our research includes a commitment to 'working with' both health professionals and patients in a process of co-design to produce outputs which we hope will reflect what really matters to staff and patients on the ground.

PATIENT AND PUBLIC INVOLVEMENT, PROJECT MANAGEMENT AND GOVERNANCE

We have a project advisory group of 11 members including academics, health professionals, representation from Age UK, 2 patient members and a lay chair. Patients have been involved in the proposal development, design of participant materials and project website (www.polypharmacy.org.uk), our application for ethical

For peer review only - http://bmjopen.bmj.com/site/about/guidelines.xhtml

approval, the project launch event, piloting of interviews, study design and conduct. We have an online patient panel of 5 members.

Site recruitment began in March 2017. Patient recruitment began January 2018 and was completed October 2018. Project funding ceases on 28/2/2021.

ETHICS AND DISSEMINATION

The project has ethics approval (IRAS project ID: 205517; REC reference 16/YH/0462).

Our findings will inform the following key outputs.

1) For clinicians: key recommendations for practice; interactive online e-learning resources
2) For professional bodies: summary of findings to inform professional guidance
3) For patients and carers: co-designed resources to inform patient and public understanding of polypharmacy, inform decision-making, and improve consultations with health professionals
4) For policymakers: a summary report of key findings
5) Academics: research publications and conference presentations

AUTHOR CONTRIBUTIONS

DS designed the study and secured funding. DS and NF have both been involved in fieldwork and have both contributed to writing this paper.

ACKNOWLEDGEMENTS

We would like to thank the staff and patients who have agreed to participate in this study. We would also like to thank members of our Expert Advisory Group and our Patient Panel for their valuable advice.

FUNDING STATEMENT

This article presents independent research funded by the National Institute for Health Research (NIHR) through a Clinician Scientist Award (DS).

In addition NF was (in part) supported by the National Institute for Health Research (NIHR) Collaboration for Leadership in Applied Health Research and Care (CLAHRC) North Thames at Bart's Health NHS Trust.

The views expressed are those of the author(s) and not necessarily those of the NHS, the NIHR or the Department of Health and Social Care.

COMPETING INTERESTS: none

For peer review only - http://bmjopen.bmj.com/site/about/guidelines.xhtml

REFERENCES

1. Masnoon N, Shakib S, Kalisch-Ellett L, Caughey GE. What is polypharmacy? A systematic review of definitions. BMC geriatrics. 2017;17(1):230.
2. Guthrie B, Makubate B, Hernandez-Santiago V, Dreischulte T. The rising tide of polypharmacy and drug-drug interactions: population database analysis 1995–2010. BMC medicine. 2015;13(1):74.
3. Avery AJ, Ghaleb M, Barber N, Franklin BD, Armstrong SJ, Serumaga B, et al. The prevalence and nature of prescribing and monitoring errors in English general practice: a retrospective case note review. The British journal of general practice : the journal of the Royal College of General Practitioners. 2013;63(613):e543-e53.
4. Sabate E. Adherence to long-term therapies: evidence for action. Geneva: World Health Organisation; 2003.
5. WHO. Adherence to long-term therapies : evidence for action. Geneva: WHO; 2003.
6. National Institute for H, Care E. Medicines adherence: Involving patients in decisions about prescribed medicines and supporting adherence. Available from: http://www.nice.org.uk/guidance/cg76/resources/guidance-medicines-adherence-pdf2009.
7. Horne R, Weinman J, Barber N, Elliott R, Morgan M, Cribb A, et al. Concordance, adherence and compliance in medicine taking: a conceptual map and research priorities. London: National Coordinating Centre for NHS Service Delivery and Organisation, 2005.
8. Charles C, Gafni A, Whelan T. Shared decision-making in the medical encounter: what does it mean?(or it takes at least two to tango). Social science & medicine. 1997;44(5):681-92.
9. Marinker M, Shaw J. Not to be taken as directed: putting concordance for taking medicines into practice. British Medical Journal Publishing Group; 2003.
10. Barnett K, Mercer SW, Norbury M, Watt G, Wyke S, Guthrie B. Epidemiology of multimorbidity and implications for health care, research, and medical education: a cross-sectional study. Lancet. 2012;380:37-43.
11. Watt G. The subversive challenges of multimorbidity. British Journal of General Practice. 2017;67(659):280-1.
12. Armstrong N, Swinglehurst D. Understanding medical overuse: the case of problematic polypharmacy and the potential of ethnography. Family Practice. 2018:cmy022-cmy.
13. Royal Pharmaceutical S. Medicines Optimisation: Helping patients to make the most of medicines. Available at: http://www.rpharms.com/promoting-pharmacy-pdfs/helping-patients-make-the-most-of-their-medicines.pdf2013.
14. National Institute for H, Care E. Medicines optimisation: the safe and effective use of medicines to enable the best possible outcomes. Available from: https://www.nice.org.uk/guidance/ng52015.
15. Duerden M, Avery T, Payne R. Polypharmacy and medicines optimisation: Making it safe and sound. London: The King's Fund; 2013.

For peer review only - http://bmjopen.bmj.com/site/about/guidelines.xhtml

16. Skrabanek P. Risk factor epidemiology: Science or non-science. Social Affairs Unit, Health, Lifestyle and Environment: Countering the Panic, London: Social Affairs Unit. 1991.
17. Heath I. Role of fear in overdiagnosis and overtreatment—an essay by Iona Heath. Bmj. 2014;349:g6123.
18. O'Sullivan JW, Stevens S, Hobbs FR, Salisbury C, Little P, Goldacre B, et al. Temporal trends in use of tests in UK primary care, 2000-15: retrospective analysis of 250 million tests. bmj. 2018;363:k4666.
19. Swinglehurst D, Fudge N. The polypharmacy challenge: time for a new script? : British Journal of General Practice; 2017.
20. Sinnott C, Mc Hugh S, Boyce MB, Bradley CP. What to give the patient who has everything? A qualitative study of prescribing for multimorbidity in primary care. The British journal of general practice : the journal of the Royal College of General Practitioners. 2015;65(632):e184-e91.
21. Webber MM, Rittel H. Dilemmas in a general theory of planning. Policy sciences. 1973;4(2):155-69.
22. Dixon-Woods M. What can ethnography do for quality and safety in health care? Quality and Safety in Health Care. 2003;12:326-7.
23. McIntosh P. Action research and reflective practice: Creative and visual methods to facilitate reflection and learning: Routledge; 2010.
24. Public Health England (PHE). National General Practice Profiles. 2019.
25. Stake RE. Qualitative Case Studies. In: Denzin NK, Lincoln YS, editors. The Sage Handbook of Qualitative Research. 3rd Sage Publications, Inc; 2005. p. 443-66.
26. Swinglehurst DF, N. APOLLO-MM: Related Projects 2019. Available from: http://www.polypharmacy.org.uk/related-projects/.
27. Wengraf T. Qualitative research interviewing : biographical narrative and semi-structured methods. London, Thousand Oaks, New Delhi: Sage Publications; 2001.
28. Gaver B, Dunne T, Pacenti E. Design: cultural probes. interactions. 1999;6(1):21-9.
29. Boehner K, Gaver W, Boucher A. Probes. Inventive Methods: Routledge; 2012. p. 199-215.
30. Wherton J, Ssugarhood P, Procter R, Rouncefield M, Dewsbury G, Hinder S, et al. Designing assisted living technologies 'in the wild': preliminary experiences with cultural probe methodology. BMC Medical Research Methodology. 2012;12:188.
31. Greenhalgh T, Wherton J, Sugarhood P, Hinder S, Procter R, Stones R. What matters to older people with assisted living needs? A phenomenological analysis of the use and non-use of telehealth and telecare. Social science & medicine. 2013;93:86-94.
32. Feldman MS, Pentland BT. Reconceptualizing Organizational Routines as a Source of Flexibility and Change. Administrative Science Quarterly. 2003;48(1):94-118.
33. Pentland BT, Feldman MS. Organizational routines as a unit of analysis. Industrial and Corporate Change. 2005;14(5):793-815.
34. Feldman MS. A performative perspective on stability and change in organizational routines. Industrial and Corporate Change. 2003;12(4):727-52.
35. Feldman MS. Organizational Routines as a Source of Continuous Change. Organization Science. 2000;11(6):611-29.

For peer review only - http://bmjopen.bmj.com/site/about/guidelines.xhtml

36. Geertz C. Thick Description: Toward an Interpretive Theory of Culture. The Interpretation of Cultures. New York: Basic Books; 1973. p. 3-30.
37. Swinglehurst D, Greenhalgh T, Russell J, Myall M. Receptionist input to quality and safety in repeat prescribing in UK general practice: ethnographic case study. BMJ. 2011;343:d6788.
38. Dionysiou DD, Tsoukas H. Understanding the (re) creation of routines from within: A symbolic interactionist perspective. Academy of Management Review. 2013;38(2):181-205.
39. Wright A. Organizational routines as embodied performatives: A communication as constitutive of organization perspective. Organization. 2016;23(2):147-63.
40. Schoeneborn D, Kuhn TR, Kärreman D. The communicative constitution of organization, organizing, and organizationality. Organization Studies. 2018:0170840618782284.
41. Cooren F, Kuhn T, Cornelissen JP, Clark T. Communication, organizing and organization: An overview and introduction to the special issue. Organization studies. 2011;32(9):1149-70.
42. Ashcraft KL, Kuhn TR, Cooren F. 1 Constitutional Amendments:"Materializing" Organizational Communication. Academy of Management annals. 2009;3(1):1-64.
43. Putnam L, Nicotera AM. Building theories of organization : the constitutive role of communication. New York ; Abingdon: Routledge; 2009.
44. Cooren F, Fairhurst GT. Dislocation and Stabilization: how to scale up from interactions to organization. In: Putnam L, Nicotera AM, editors. Building Theories of Organization: the constitutive role of communication. New York, London: Routledge; 2009. p. 117-52.
45. Boje DM. Storytelling organizations: Sage; 2008.
46. Gabbay J, le May A. Evidence based guidelines or collectively contructed "mindlines?" Ethnographic study of knowledge management in primary care. BMJ. 2004;329 1013-7.
47. Czarniawska B. A theory of organizing. 2nd ed: Edward Elgar Publishing; 2014.
48. Iedema R. Creating safety by strengthening clinicians' capacity for reflexivity. BMJ quality & safety. 2011;20(Suppl 1):i83-i6.
49. Iedema R, Mesman J, Carroll K. Visualising Health Care Practice Improvement: Innovation From Within. London, New York: Radcliffe Publishing Ltd; 2013.
50. Iedema R, Carroll K, Collier A, Hor S, Mesman J, Wyer M. Video-reflexive ethnography in health research and healthcare improvement. Boca Raton, London, New York: CRC Press; 2019.
51. Iedema R, Carroll K. Discourse research that intervenes in the quality and safety of care practices. Discourse & Communication. 2010;4(1):68-86.
52. Schon DA. The reflective practitioner: How professionals think in action. New York: Basic Books Inc; 1983.
53. Bate P, Robert G. Experience-based design: from redesigning the system around the patient to co-designing services with the patient. Quality and Safety in Health Care. 2006;15(5):307-10.
54. Bate P, Robert G. Bringing user experience to healthcare improvement: The concepts, methods and practices of experience-based design: Radcliffe Publishing; 2007.

For peer review only - http://bmjopen.bmj.com/site/about/guidelines.xhtml

55. Thomson A, Rivas C, Giovannoni G. Multiple sclerosis outpatient future groups: improving the quality of participant interaction and ideation tools within service improvement activities. BMC Health Service Research. 2015;15(105).
56. Nicolini D. Practice Theory, Work, & Organization: An introduction. Oxford: Oxford University Press; 2012.
57. Nicolini D. Zooming in and zooming out: A package of method and theory to study work practice. In: Ybema S, Yanow D, Wels H, Kamsteeg F, editors. Organizational Ethnography. London, Thousand Oaks, New Delhi: Sage Publications; 2009. p. 120-38.

For peer review only

For peer review only - http://bmjopen.bmj.com/site/about/guidelines.xhtml

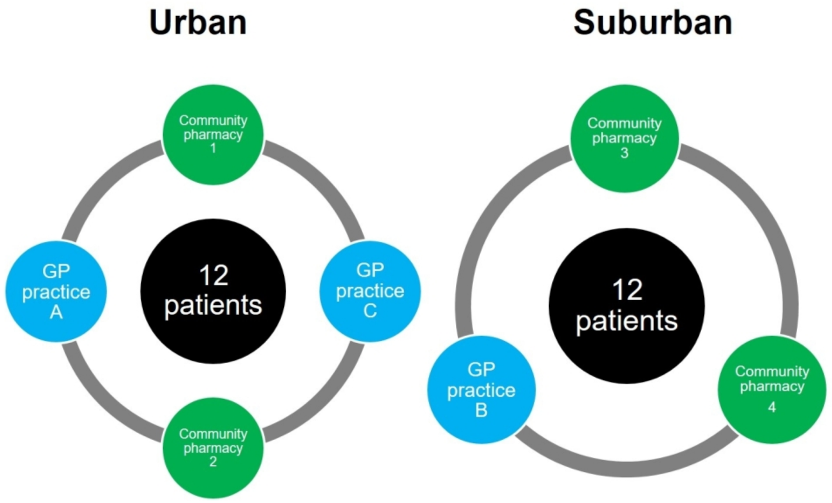

Figure 1. Study sites and research clusters

287x157mm (150 x 150 DPI)

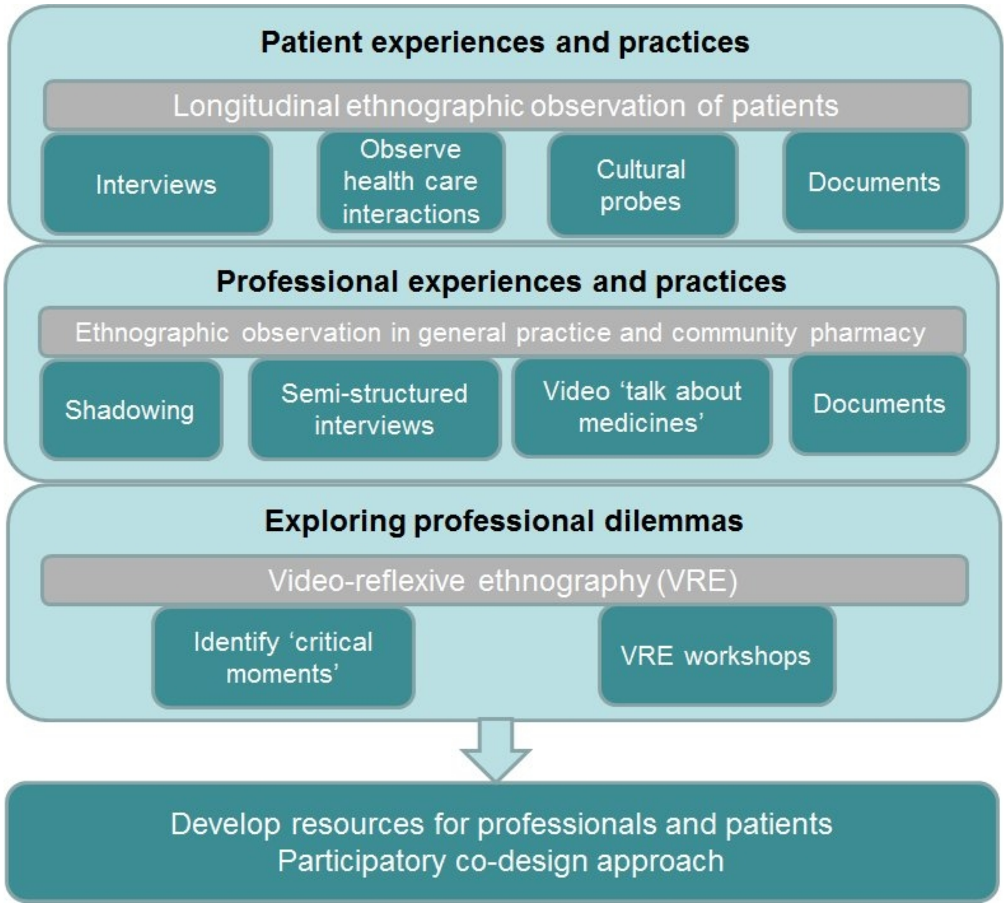

Figure 2: Methods and data sources

277x249mm (72 x 72 DPI)

BMJ Open

Addressing the Polypharmacy Challenge in Older People with Multimorbidity (APOLLO-MM): study protocol for an in-depth ethnographic case study in primary care

Journal:	*BMJ Open*
Manuscript ID	bmjopen-2019-031601.R1
Article Type:	Protocol
Date Submitted by the Author:	15-Jul-2019
Complete List of Authors:	Swinglehurst, Deborah; Queen Mary University of London Faculty of Medicine and Dentistry, Centre for Primary Care and Public Health<br>Fudge, Nina ; Queen Mary University of London Faculty of Medicine and Dentistry, Centre for Primary Care and Public Health
**Primary Subject Heading**:	Qualitative research
Secondary Subject Heading:	Patient-centred medicine, Sociology, Research methods
Keywords:	polypharmacy, multimorbidity, ethnography, PRIMARY CARE

SCHOLARONE™
Manuscripts

TITLE: Addressing the Polypharmacy Challenge in Older People with Multimorbidity (APOLLO-MM): study protocol for an in-depth ethnographic case study in primary care

Authors: Deborah Swinglehurst*, Nina Fudge

*Corresponding author

CONTACT DETAILS

Professor Deborah Swinglehurst

Centre for Primary Care and Public Health

Blizard Institute

Barts and The London School of Medicine and Dentistry

Queen Mary University of London

Yvonne Carter Building

58 Turner Street

London E1 2AB

UK

Telephone: +44 (0)20 7882 7204

E mail: d.swinglehurst@qmul.ac.uk

ORCID ID: https://orcid.org/0000-0003-1261-9268

Dr Nina Fudge (same institutional address as DS)

Telephone: +44 (0)20 7882 2528

E mail: n.fudge@qmul.ac.uk

ORCID ID: http://orcid.org/0000-0002-7161-4355

WORD COUNT: 3992

For peer review only - http://bmjopen.bmj.com/site/about/guidelines.xhtml

ABSTRACT

Introduction

Polypharmacy is on the rise. It is burdensome for patients and is a common source of error and adverse drug reactions, especially amongst older adults. Health policy advises clinicians to practice *medicines optimisation* – a person-centred approach to safe, effective medicines use. There has been little research exploring older patients' perspectives and priorities around medicines-taking, nor their actual practices of fitting medicines into their daily lives and how these are shaped by the wider context of health care.

Methods and Analysis

We will conduct an in-depth multisite ethnographic case study. The study is based in 7 clinical sites (3 general practices; 4 community pharmacies) and includes longitudinal ethnographic follow-up of older adults, organisational ethnography and participatory methods. Main data sources include: field notes of observations in the home and clinical settings; interviews with patients and professionals; cultural probe activities; video-recordings of clinical consultations and inter-professional talk; documents. Our analysis will illuminate: the everyday practices of polypharmacy from a range of lay and professional perspectives; the institutional contexts within which these practices play out; and the sense-making work that sustains - or challenges - these practices. Our research will adopt a 'practice theory' lens, drawing on the sociology of organisational routines, and other relevant social theory guided by ongoing iterative data analysis.

Ethics Approval

The study has HRA approval and received a favourable ethical opinion from the Leeds West Research Ethics Committee (IRAS project ID: 205517; REC reference 16/YH/0462).

Dissemination

Aside from academic outputs, our findings will inform the development of recommendations for practice and policy including an interactive e-learning resource. We will also work with service users to co-design patient/public engagement resources.

For peer review only - http://bmjopen.bmj.com/site/about/guidelines.xhtml

Keywords: polypharmacy; multimorbidity; ethnography; primary care

ARTICLE SUMMARY: STRENGTHS AND LIMITATIONS OF THIS STUDY

- Gathers rich qualitative data across community settings (the home; general practice; pharmacy) with a focus on how practices unfold on the ground and how different actors make sense of polypharmacy
- Brings together analysis of patients' lived experience with analysis of organisational routines and professional practices
- Uses innovative qualitative methodologies which generate more sophisticated insights than is possible with conventional approaches
- Prioritises depth of analysis over breadth, increasing richness of understanding but limiting generalisability of findings.

INTRODUCTION

Polypharmacy is increasing. One Scottish study showed that about 20% of adults are dispensed five or more drugs (this doubled between 1995 and 2010) and about 6% of adults are dispensed 10 or more drugs (this tripled over the same time period) (1). There is growing concern that polypharmacy is one example of 'medical overuse', causing unnecessary burden, iatrogenic harm to patients and costly waste to health systems. Up to half of medicines prescribed for long term conditions are unused (2). This is often conceptualised as a problem of poor adherence (3-5) or as a failure of shared decision-making in the consultation (6, 7). The problem is assumed to be located within individuals' behaviours, with relatively little attention paid to the relationship between individuals and the wider social, organisational and institutional context within which their medicines practices play out.

Polypharmacy is often attributed to the ageing population; 65% of people aged 65 – 84, and over 80% of people aged 85+ experience multimorbidity (8). But ageing *per se* is only one of many potentially relevant factors. For example, evidence-based guidance which informs prescribing is typically organised around a 'single disease' model and may compound rather than alleviate polypharmacy. One critic has described evidence based medicine as a perverse 'monument to bias', built on its systematic exclusion of complex patients with multimorbidity from the trials that inform it (9). Arguably polypharmacy in the context of multimorbidity is rarely, if ever, evidence-based, even when a clear 'evidence-based' argument can be made for individual items (10). Achieving *medicines optimisation* – a person-centred approach to safe, effective medicines use (11-13) - is fraught with difficulty when the institutional context privileges the single disease model and is increasingly concerned with the application of abstract, generalisable rules. This is further complicated by the increasing emphasis on what has been called 'risk factorology' and the quest to eliminate risk of disease altogether (14).The societal context is

For peer review only - http://bmjopen.bmj.com/site/about/guidelines.xhtml

increasingly one in which "every responsible and rational citizen is expected to actively seek out and eliminate all possible risks to their future health and to consume medical technologies in order to achieve this aim" (15). Pay-for-performance initiatives which embrace (and arguably constitute) aspirations to eliminate risk and uncertainty may also drive polypharmacy.

A recent study which examined temporal trends between 2000 and 2015 in the use of tests and investigations in UK primary care found that age- and sex- adjusted use of tests and investigations increased by a staggering 8.5% *annually* (16). Although prevalence studies do not offer insight into the drivers of change, it seems reasonable to conclude that the parallel and dramatic rise in investigations and polypharmacy over a similar time period may be related phenomena. Privileging priorities for reducing risk of disease may contribute paradoxically to new drug-related risks – risks which increase with increasing age and with increasing numbers of medications (17). 'Problematic' polypharmacy (13) arises insidiously, despite (and sometimes even because of) clinicians and patients striving to achieve best practice and acting with the very best of intentions. The elusive boundary between 'just enough medicine' and 'too much medicine' is unstable, contingent and socially constructed. It involves clinical judgement in the context of inadequate evidence and considerable uncertainty. Sinnott has referred to the work of negotiating this boundary as a process of 'satisficing' (18). Doctors report settling for management that is satisfactory and sufficient given the particular circumstances of a patient - a combination of relaxing nationally recommended targets, compromise, hunch, guesswork and maintaining the status quo when patients appear stable (18).

Polypharmacy presents a complex challenge for clinicians and health systems. It arises at the interface of patients, clinicians, diseases (and risk thereof) and involves a tangle of biological, cultural, technological, economic and socio-political dimensions. It is a 'wicked problem' (19). One feature of wicked problems is their resistance to adequate definition. Polypharmacy tends to be defined numerically, with one recent systematic review identifying 138 different definitions (20). But simple numerical definitions conceal much of the complexity that the polypharmacy concept evokes (17). Rittel states that "the formulation of a wicked problem *is* the problem"; wicked problems are ill-defined, not amenable to a quick-fix 'solutions' but at best '*resolved, or rather re-solved, over and over again*'. Wicked problems coalesce around important moral issues whereby there are acknowledged discrepancies between the state of affairs as it *is* and the state as it *ought* to be (19). Ethnographic approaches are especially good at investigating complex issues such as polypharmacy and shedding light on ill-defined but troubling phenomena (21). In this study we will adopt an ethnographic approach and apply a range of innovative methods which focus on meanings, experiences and practices as we encounter them in the lives of patients and professionals. This approach embraces polypharmacy as a wicked problem, offering 'new ways of looking' and thereby opening up potential new avenues for addressing polypharmacy in practice and policy.

Aim

For peer review only - http://bmjopen.bmj.com/site/about/guidelines.xhtml

The aim of the APOLLO-MM project is to improve patient care by producing 'practice-based' evidence (22) to inform medicines optimisation. We will do this by:

- Examining patients' experiences of polypharmacy to discern elements of high quality 'medicines work' and those elements that might lead to harmful, wasteful or unnecessary polypharmacy
- Improving understanding of how polypharmacy is sustained and/or challenged within and between lay and professional networks
- Analysing consultations that include talk about medicines (including but not limited to formal 'medication review') to understand how medicine optimisation is negotiated in practice
- Using participatory methods to elicit professional dialogue around polypharmacy and offer opportunities to discern the elements of good practice in medicines optimisation
- Working with patients and professionals in a process of co-design to develop e-learning materials, recommendations for practice and public/patient engagement resources

Research questions

1. What is the patient experience of polypharmacy in multimorbidity?
2. What does polypharmacy mean for patients and carers?
    a. How are patients' lives with multimorbidity shaped by practices of managing medicines?
    b. How is the managing of medicines shaped by contingencies of living with multimorbidity?
3. How do the practices of patients / carers / health professionals and wider systems of care support 'appropriate' polypharmacy or challenge 'inappropriate' polypharmacy and with what consequences for the quality and safety of care?
4. How can insights from longitudinal patient case studies inform improvements in professional practices, service delivery and public awareness of polypharmacy?

METHODS

Setting and Context

The study is a multicentre ethnographic case study involving seven clinical sites across two research clusters in England (one urban and one suburban cluster). The urban cluster comprises two NHS GP practices (Site A, Site C) and two independent community pharmacies located in an area of high socio-economic deprivation within a large city, serving a population of diverse ethnicity comprising approximately 40% Asian patients. The suburban cluster comprises one NHS GP practice (Site B) and two independent community pharmacies, located in a small, relatively prosperous city serving a population which is 85% white Caucasian. The research clusters represent contrasting demographics; the urban cluster serves patient populations within the lowest (Site C) and second lowest (Site A) decile of the English Index of multiple deprivation score (23), and the suburban cluster serves a population within the least deprived decile.

For peer review only - http://bmjopen.bmj.com/site/about/guidelines.xhtml

The GP practices were selected following expressions of interest from over forty GP practices within the North Thames Clinical Research Network area, suggesting that the project resonates with the concerns of GPs on the ground.

We have recruited 24 patients, 14 in the urban cluster (7 each from site A and C) and 10 from our suburban cluster. The work of patient follow-up and organisational ethnography has begun. The GP practices and community pharmacies in the study share responsibility for prescribing and dispensing medicines to the study patients, allowing for multiple perspectives of polypharmacy. To date we have recruited 40 professional participants for interviews, filming of consultations and participation in research workshops (see section on *Study Design, Methodology and Approach to Analysis*). The relationship between the patients, GP practices and community pharmacies is shown in Figure 1.

[INSERT FIGURE 1 ABOUT HERE] Figure 1. Study sites and research clusters

Selection of organisational cases

Drawing on Stakes approach to organisational case study our selection of GP sites primarily reflects 1) *opportunity for learning*, rather than concerns about representativeness (24) and 2) diversity of geographical setting and population demographics. Our concern is to conduct an in-depth study of *particular* practices within these sites, adopting an interpretive approach informed by relevant social theory. We will create generalisable theoretical and conceptual abstractions (novel concepts to 'think with') rather than generalisable statistical abstractions. The ethnographic approach relies on the interest shown in the study by the potential participants, the willingness of staff at all levels of the organisation to allow meaningful access to the research team (e.g. shadowing, observations, formal and informal interviews; access to relevant documents) and enthusiasm for the participatory element of the study within the context of already heavy workloads (see *Study Design*). In addition we considered a range of pragmatic concerns relating to feasibility, location and likelihood of building sustainable working relationships for the duration of the study. Table 1 shows some of the characteristics of our recruited practices.

[INSERT TABLE 1 ABOUT HERE]

For peer review only

Table 1. Characteristics of recruited GP practices

	Site A	Site B	Site C
Cluster	Urban	Suburban	Urban
Patient population	c.11000	c.13000	c.14000
General Practitioners	5 partners; 5 salaried	4 partners; 6 salaried	8 partners; 3 salaried
On-site clinical pharmacist at recruitment	No	No	No
On-site clinical pharmacist for part of project duration	Yes	Yes	Yes
GP training practice	Yes	Yes	Yes
Deprivation*	2nd most deprived decile	Least deprived decile	Most deprived decile
Ethnic diversity (estimated proportion non-white ethnic groups)*	Over 50% (over 40% of practice population are Asian)	14%	Over 60% (over 40% of practice population are Asian)

**details from National General Practice Profiles produced by Public Health England Data Science et al. (REF)*

Sampling of patient participants

We have recruited patients aged 65 or over and prescribed 10 or more regular (repeat) items of medication, this being regarded a pragmatic marker of 'high risk polypharmacy' (13). Where patients were prescribed an item in more than one dose (e.g. warfarin), this was counted as a single item. Medical devices and hosiery were excluded, but medicines in all forms (e.g. oral, inhalers, injectables, topical creams etc) were included. We adopted a purposive sampling approach, aiming for a maximum diversity sample across a range of attributes: age (ranging from 65 – 94); gender (11 men; 13 women); living circumstances (16 live alone of whom 5 in sheltered housing; 8 with partners/family; 4 housebound); socioeconomic status (home ownership and previous occupation as proxy indicators); number of medication items (10 – 17). All patients have two or more comorbidities. 21 of 24 patients are white Caucasian. We have excluded patients unable to speak adequate English, which means that sampling in our urban cluster is not representative of the local population. We are addressing this limitation in another study (25).

Recruitment took place in general practice. GPs and nurses introduced the study to potential participants, issued participant information leaflets and sought verbal consent from patients to be contacted by a researcher (n=63). 36 patients agreed to a home visit to discuss the study in more detail and 24 consented to take part. Patients lacking capacity to consent were eligible for inclusion on the identification of a personal consultee. A consultee is someone who, by virtue of their existing relationship with the potential participant, can advise the researcher about their participation in the project. One patient (with dysphasia following a stroke) has a personal consultee, their next of kin who is their primary caregiver. We were unable to recruit any patients with dementia; we are addressing this using a different recruitment strategy in a parallel study which focusses specifically on patients with dementia (25)

For peer review only - http://bmjopen.bmj.com/site/about/guidelines.xhtml

STUDY DESIGN, METHODOLOGY AND APPROACH TO ANALYSIS

Figure 2 shows the methods and data sources for the study, which are designed to elicit and explore patient experiences and practices; professional experiences and practices; professional dilemmas. These are described in detail in the following sections.

[INSERT FIGURE 2 ABOUT HERE] Figure 2: Methods and data sources

Patient experiences and practices

We will follow patient participants ethnographically over 18-24 months, observing them at home and accompanying them to selected health care interactions in which they anticipate talk about their medicines (e.g. at the GP surgery, pharmacy or hospital). We are conducting interviews with patients to elicit the story of their lives since they were first advised to take medicines, adapting Wengraf's Biographic-Narrative Interpretive Method (BNIM) (26). This approach utilises a single question to induce narrative (the SQUIN) and invites participants to speak uninterrupted until their story is complete. After a short break (10 – 15 minutes) the researcher presents a series of further questions based on 'cue phrases' (the patient's own words) in the order the patient introduced them into their opening narrative, to encourage further in-depth narratives (particular incident narratives or PINs). On a separate occasion we return to the patient's home to conduct an in-depth interview guided by a topic guide with a more explicit focus on medicines practices. We contact participants by telephone or home visit at approximately 4 – 8 weekly intervals to learn about their ongoing experiences and to identify occasions when we may accompany them to a health care interaction or video-record a consultation. Every time we meet our participants we take detailed ethnographic field notes, add to our reflexive journal and gather relevant documents (e.g. discharge summary or lists of medication). This data enables us to build detailed patient case studies as we follow their lived experience and engage them in informal 'event based' interviews exploring their care experiences.

A subset of patients are completing cultural probe activities as a way of depicting their lives. Cultural probes originated in the world of design as a way of tapping into peoples' creativity and offering research participants opportunity to engage in a research-related activity that is subjective, deliberately ambiguous, open to interpretation, invites imagination and stimulates conversation (27, 28). The probes consist of a set of activities and a range of materials (e.g. camera, pens, cards, booklets) with simple instructions which offer opportunity for improvisation. They encourage participants to think about their medicines and bodies in new ways and re-imagine possibilities for how they might interact with health professionals. The creative outputs are used to stimulate an informal discussion between researcher and participant. Participants may invite a family member or researcher to assist if they wish. Cultural probes have been used successfully to incorporate older people's ideas into the design of social communities (27), and to illuminate the daily

For peer review only - http://bmjopen.bmj.com/site/about/guidelines.xhtml

living needs of older people to inform co-production of assistive living technologies (29, 30) although it is recognised that some people may be unable to engage with them for a range of physical, emotional and social reasons (29).

Professional experiences and practices

We will conduct ethnographic observations in three GP and four community pharmacy practices (see Figure 1). Our interest is in understanding how health professionals conduct and make sense of their day-to-day work of prescribing, dispensing, negotiating, organising medicines for patients affected by polypharmacy. We will observe key routines such as: preparation and organisation of multicompartment compliance aids (MCAs or 'dosette boxes'); repeat prescribing and repeat dispensing.

We will closely observe: individuals at work (shadowing, and asking participants to describe what they are doing as they do it to encourage the articulation of tacit knowledge); organisational routines and how they are constituted; the wider institutional context; documents relevant to the work we observe (e.g. prescriptions, guidelines). We will also interview key professional participants across our sites with a focus on accounts of their working practices. We will hone in on consultations between clinicians and our study patients to examine healthcare interactions in detail by video-recording consultations in which our participants anticipate 'talk about medicines'. The polypharmacy context is a very particular context for these interactions and we are interested to learn how polypharmacy is sustained, challenged, talked about and 'brought about' in these interactions.

Organisational routines are conceptualised as "repetitive recognisable patterns of interdependent actions by multiple actors" (31, 32) and can be regarded as sites of stability and as sites of potential for innovation and change (33). When participants engage in routines they enact tacit knowledge, and are involved in complex patterns of collaboration – involving people, artefacts, technologies - to get work done. Routines can be embodied, embraced, resisted, and enacted with varying degrees of creativity and improvisation, and varying attention to the particularities of local context (34). Pentland and Feldman have conceptualised routines as being comprised of:

- *ostensive routines* - the generalised 'rules' or organisational scripts that guide the routine, of which there may be many instantiations.
- *performative routines* - what people *actually do in practice* as they engage in the routine. Each instantiation of the routine is a unique performance.

Ostensive routines may be accessed through peoples' accounts of the work they do whilst observation of repeated rounds of a routine gives insight into the many versions of the performative routine and the relationship between the performative and the ostensive aspects of the routines. When people engage in routines they select from a repertoire of possible actions (enacting possibilities around which there are explicit or implicit constraints). Different people bring different knowledge, different expertise, different assumptions and different personal and organisational narratives to the enactment of the routine. An understanding of the patterns of

For peer review only - http://bmjopen.bmj.com/site/about/guidelines.xhtml

possibility and constraints offers insights into wider organisation principles and values, and to an appreciation of complex organisational practices, such as how power circulates and how learning is done. The dynamic interplay between the ostensive and performative routines and the artefacts which are drawn upon to support these routines can transform what has traditionally been regarded as mundane and ordinary organisational life (e.g. the processing of a repeat prescription) into opportunity for 'thick description' (35) of the nature of organisational life and the wider context within which work is being done (32, 36). Continuities and discontinuities in the relationship between ostensive and performative routines may become manifest as puzzles, conflicts, moments of perplexity or tensions.

Recent literature on organisational routines has pointed to the need to pay greater attention to the relational nature of 'self' and agency in the performance of routines and in the dynamic processes that address *how routines become collective accomplishments* with recognisable shared collective characteristics (i.e. how the 'ostensive' aspect of routines is constituted or brought about) (37, 38). One approach which offers promise is to locate the study of routines within emerging 'communication as constitutive of organisation (CCO)' scholarship (39-42) wherein communication is itself framed as performance (through conversation and text dialectic). Routines are then framed as communicatively constituted performances, there is greater emphasis placed on performativity, embodiment, dialogic interrelationships, and interest in 'strings of associations' that link actors (human and non-human) as routines unfold (43). The nuance of communication gains ground - the emergence of organisational storylines, for example (44-46). This approach aligns with our interest in patient narratives and our orientation to 'talk about medicines' as co-constructed accomplishment within the consultation.

Video Reflexive Ethnography (VRE)

Video-reflexive ethnography is an innovative approach to quality and safety research in health care (47-49) which encourages groups of professionals to discuss and reflect on their working practice. In this study, selected video-recorded footage of 'medicines talk' in our study patients' consultations will be shown to small groups of professionals across our research sites. VRE offers an opportunity for participants to negotiate shared meanings in their practice, engage with the complexity of their everyday work, and make visible - in new ways - aspects of their practice which have become habituated, taken-for- granted, and 'invisible' (49). It is a participatory approach in which researchers and practitioners work together to illuminate this complexity and foster dialogue that might not otherwise occur (50). The footage acts as a catalyst, making implicit knowledge explicit through a process of reflection-on-action which renders the work (in this case the practical work of 'medicines optimisation') understandable in new ways and open to new ways of re-imagining practice (51). Importantly the discussion may go beyond what is visible in the footage, the footage acting as a prompt to inter-professional exchange and peer-led discussion of professional concerns, dilemmas and opportunities. There is opportunity for 'exnovation' or innovation from *within* practice (48) and the cultivation of meta-discourses of practice (50) which can reframe everyday practices (for example the 'medication review') by drawing out the systemic implications of what

For peer review only - http://bmjopen.bmj.com/site/about/guidelines.xhtml

specific people know, do and say. By video-recording the discussion this meta-discourse can be teased out and worked with across VRE sessions, in ways which enable practitioners to iteratively reframe their work and negotiate new possibilities for action. Importantly the collaborative process of co-constructing this meta-discourse in the VRE context may itself be a rehearsal space for new ways of relating and collaborating in the very doing of the work that is under scrutiny.

Participatory co-design

The findings from the research will feed into a process of participatory co-design, working in collaboration with both patients and professionals to design e-learning materials for professional education and patient engagement resources for patients. In this work we will adapt processes of experience-based co-design (52, 53) and 'future groups' (54), the latter being a reinterpretation of focus group methods which uses ideation tools and encourages speculation to imagine alternative approaches to care. We will collaborate with a design researcher to develop our research materials and findings to create bespoke ideation tools and props to facilitate imaginative participation and move towards exploring future alternatives to care prompted by the stories, data and findings from the earlier research.

Integrating analysis across diverse datasets

The project is situated within a broad 'practice theory' orientation and adopts a social constructionist orientation. Practice theory encourages an understanding of work as an accomplishment – and fosters a curiosity about what is accomplished by whom and how. The organisation (e.g. the GP practice or pharmacy) is understood as both the *site* and the *result* of work activities and the role of the interpretive researcher is to 'zoom in' on the details of particular practices and to 'zoom out' to discern the relationships, and connections between people, routines, artefacts, spaces and technologies for example (55, 56). As our analysis progress we will, in addition draw, on relevant social theory (e.g. Burden of Treatment theory (57, 58)) to explicate our findings.

This detailed ethnography will extend our previous research on repeat prescribing in general practice settings in four ways (36). Firstly the institutional context has changed (one example being the implementation of the Electronic Prescribing Service, another being the incorporation of clinical pharmacists within some GP settings). Secondly, our research extends beyond general practice into community pharmacy settings and into peoples' homes and opens up a wider range of vantage points from which to observe the phenomenon. We are focussing our observations around the phenomenon of polypharmacy, recognising that polypharmacy emerges out of an arrangement of practices and is not a discrete entity that can be easily observed without appreciation of a wider social context. Finally our research includes a commitment to 'working with' both health professionals and patients in a process of co-design to produce outputs which we hope will reflect what really matters to staff and patients on the ground.

For peer review only - http://bmjopen.bmj.com/site/about/guidelines.xhtml

PATIENT AND PUBLIC INVOLVEMENT

We have a project advisory group of 11 members including academics, health professionals, representation from Age UK, 2 patient members and a lay chair. Patients have been involved in the proposal development, design of participant materials and project website (www.polypharmacy.org.uk), our application for ethical approval, the project launch event, piloting of interviews, study design and conduct. We have an online patient panel of 5 members.

PROJECT MANAGEMENT AND GOVERNANCE

Our project is overseen by an expert advisory group (see above) and we report annually to the Heath Research Authority and our funder (National Institute of Health Research).

Site recruitment began in March 2017. Patient recruitment began January 2018 and was completed October 2018. Project funding ceases on 28/2/2021.

ETHICS AND DISSEMINATION

The project has ethics approval (IRAS project ID: 205517; REC reference 16/YH/0462).

Our findings will inform the following key outputs.

1) For clinicians: key recommendations for practice; interactive online e-learning resources
2) For professional bodies: summary of findings to inform professional guidance
3) For patients and carers: co-designed resources to inform patient and public understanding of polypharmacy, inform decision-making, and improve consultations with health professionals
4) For policymakers: a summary report of key findings
5) Academics: research publications and conference presentations

AUTHOR CONTRIBUTIONS

DS designed the study and secured funding. DS and NF have both been involved in fieldwork and have both contributed to writing this paper.

ACKNOWLEDGEMENTS

We would like to thank the staff and patients who have agreed to participate in this study. We would also like to thank members of our Expert Advisory Group and our Patient Panel for their valuable advice.

FUNDING STATEMENT

For peer review only

This article presents independent research funded by the National Institute for Health Research (NIHR) through a Clinician Scientist Award (DS).

In addition NF was (in part) supported by the National Institute for Health Research (NIHR) Collaboration for Leadership in Applied Health Research and Care (CLAHRC) North Thames at Bart's Health NHS Trust.

The views expressed are those of the author(s) and not necessarily those of the NHS, the NIHR or the Department of Health and Social Care.

COMPETING INTERESTS: none

For peer review only

REFERENCES

1. Guthrie B, Makubate B, Hernandez-Santiago V, Dreischulte T. The rising tide of polypharmacy and drug-drug interactions: population database analysis 1995–2010. BMC medicine. 2015;13(1):74.
2. Sabate E. Adherence to long-term therapies: evidence for action. Geneva: World Health Organisation; 2003.
3. WHO. Adherence to long-term therapies : evidence for action. Geneva: WHO; 2003.
4. National Institute for H, Care E. Medicines adherence: Involving patients in decisions about prescribed medicines and supporting adherence. Available from: http://www.nice.org.uk/guidance/cg76/resources/guidance-medicines-adherence-pdf2009.
5. Horne R, Weinman J, Barber N, Elliott R, Morgan M, Cribb A, et al. Concordance, adherence and compliance in medicine taking: a conceptual map and research priorities. London: National Coordinating Centre for NHS Service Delivery and Organisation, 2005.
6. Charles C, Gafni A, Whelan T. Shared decision-making in the medical encounter: what does it mean?(or it takes at least two to tango). Social science & medicine. 1997;44(5):681-92.
7. Marinker M, Shaw J. Not to be taken as directed: putting concordance for taking medicines into practice. British Medical Journal Publishing Group; 2003.
8. Barnett K, Mercer SW, Norbury M, Watt G, Wyke S, Guthrie B. Epidemiology of multimorbidity and implications for health care, research, and medical education: a cross-sectional study. Lancet. 2012;380:37-43.
9. Watt G. The subversive challenges of multimorbidity. British Journal of General Practice. 2017;67(659):280-1.
10. Armstrong N, Swinglehurst D. Understanding medical overuse: the case of problematic polypharmacy and the potential of ethnography. Family Practice. 2018:cmy022-cmy.
11. Royal Pharmaceutical S. Medicines Optimisation: Helping patients to make the most of medicines. Available at: http://www.rpharms.com/promoting-pharmacy-pdfs/helping-patients-make-the-most-of-their-medicines.pdf2013.
12. National Institute for H, Care E. Medicines optimisation: the safe and effective use of medicines to enable the best possible outcomes. Available from: https://www.nice.org.uk/guidance/ng52015.
13. Duerden M, Avery T, Payne R. Polypharmacy and medicines optimisation: Making it safe and sound. London: The King's Fund; 2013.

For peer review only - http://bmjopen.bmj.com/site/about/guidelines.xhtml

14. Skrabanek P. Risk factor epidemiology: Science or non-science. Social Affairs Unit, Health, Lifestyle and Environment: Countering the Panic, London: Social Affairs Unit. 1991.
15. Heath I. Role of fear in overdiagnosis and overtreatment—an essay by Iona Heath. Bmj. 2014;349:g6123.
16. O'Sullivan JW, Stevens S, Hobbs FR, Salisbury C, Little P, Goldacre B, et al. Temporal trends in use of tests in UK primary care, 2000-15: retrospective analysis of 250 million tests. bmj. 2018;363:k4666.
17. Swinglehurst D, Fudge N. The polypharmacy challenge: time for a new script? : British Journal of General Practice; 2017.
18. Sinnott C, Mc Hugh S, Boyce MB, Bradley CP. What to give the patient who has everything? A qualitative study of prescribing for multimorbidity in primary care. The British journal of general practice : the journal of the Royal College of General Practitioners. 2015;65(632):e184-e91.
19. Webber MM, Rittel H. Dilemmas in a general theory of planning. Policy sciences. 1973;4(2):155-69.
20. Masnoon N, Shakib S, Kalisch-Ellett L, Caughey GE. What is polypharmacy? A systematic review of definitions. BMC geriatrics. 2017;17(1):230.
21. Dixon-Woods M. What can ethnography do for quality and safety in health care? Quality and Safety in Health Care. 2003;12:326-7.
22. McIntosh P. Action research and reflective practice: Creative and visual methods to facilitate reflection and learning: Routledge; 2010.
23. Public Health England (PHE). National General Practice Profiles. 2019.
24. Stake RE. Qualitative Case Studies. In: Denzin NK, Lincoln YS, editors. The Sage Handbook of Qualitative Research. 3rd Sage Publications, Inc; 2005. p. 443-66.
25. Swinglehurst DF, N. APOLLO-MM: Related Projects 2019. Available from: http://www.polypharmacy.org.uk/related-projects/.
26. Wengraf T. Qualitative research interviewing : biographical narrative and semi-structured methods. London, Thousand Oaks, New Delhi: Sage Publications; 2001.
27. Gaver B, Dunne T, Pacenti E. Design: cultural probes. interactions. 1999;6(1):21-9.
28. Boehner K, Gaver W, Boucher A. Probes. Inventive Methods: Routledge; 2012. p. 199-215.
29. Wherton J, Ssugarhood P, Procter R, Rouncefield M, Dewsbury G, Hinder S, et al. Designing assisted living technologies 'in the wild': preliminary experiences with cultural probe methodology. BMC Medical Research Methodology. 2012;12:188.
30. Greenhalgh T, Wherton J, Sugarhood P, Hinder S, Procter R, Stones R. What matters to older people with assisted living needs? A phenomenological analysis of the use and non-use of telehealth and telecare. Social science & medicine. 2013;93:86-94.
31. Feldman MS, Pentland BT. Reconceptualizing Organizational Routines as a Source of Flexibility and Change. Administrative Science Quarterly. 2003;48(1):94-118.
32. Pentland BT, Feldman MS. Organizational routines as a unit of analysis. Industrial and Corporate Change. 2005;14(5):793-815.
33. Feldman MS. A performative perspective on stability and change in organizational routines. Industrial and Corporate Change. 2003;12(4):727-52.
34. Feldman MS. Organizational Routines as a Source of Continuous Change. Organization Science. 2000;11(6):611-29.
35. Geertz C. Thick Description: Toward an Interpretive Theory of Culture. The Interpretation of Cultures. New York: Basic Books; 1973. p. 3-30.
36. Swinglehurst D, Greenhalgh T, Russell J, Myall M. Receptionist input to quality and safety in repeat prescribing in UK general practice: ethnographic case study. BMJ. 2011;343:d6788.
37. Dionysiou DD, Tsoukas H. Understanding the (re) creation of routines from within: A symbolic interactionist perspective. Academy of Management Review. 2013;38(2):181-205.
38. Wright A. Organizational routines as embodied performatives: A communication as constitutive of organization perspective. Organization. 2016;23(2):147-63.

For peer review only - http://bmjopen.bmj.com/site/about/guidelines.xhtml

39. Schoeneborn D, Kuhn TR, Kärreman D. The communicative constitution of organization, organizing, and organizationality. Organization Studies. 2018:0170840618782284.
40. Cooren F, Kuhn T, Cornelissen JP, Clark T. Communication, organizing and organization: An overview and introduction to the special issue. Organization studies. 2011;32(9):1149-70.
41. Ashcraft KL, Kuhn TR, Cooren F. 1 Constitutional Amendments:“Materializing” Organizational Communication. Academy of Management annals. 2009;3(1):1-64.
42. Putnam L, Nicotera AM. Building theories of organization : the constitutive role of communication. New York ; Abingdon: Routledge; 2009.
43. Cooren F, Fairhurst GT. Dislocation and Stabilization: how to scale up from interactions to organization. In: Putnam L, Nicotera AM, editors. Building Theories of Organization: the constitutive role of communication. New York

London: Routledge; 2009. p. 117-52.
44. Boje DM. Storytelling organizations: Sage; 2008.
45. Gabbay J, le May A. Evidence based guidelines or collectively contructed "mindlines?" Ethnographic study of knowledge management in primary care. BMJ. 2004;329 1013-7.
46. Czarniawska B. A theory of organizing. 2nd ed: Edward Elgar Publishing; 2014.
47. Iedema R. Creating safety by strengthening clinicians' capacity for reflexivity. BMJ quality & safety. 2011;20(Suppl 1):i83-i6.
48. Iedema R, Mesman J, Carroll K. Visualising Health Care Practice Improvement: Innovation From Within. London, New York: Radcliffe Publishing Ltd; 2013.
49. Iedema R, Carroll K, Collier A, Hor S, Mesman J, Wyer M. Video-reflexive ethnography in health research and healthcare improvement. Boca Raton

London

New York: CRC Press; 2019.
50. Iedema R, Carroll K. Discourse research that intervenes in the quality and safety of care practices. Discourse & Communication. 2010;4(1):68-86.
51. Schon DA. The reflective practitioner: How professionals think in action. New York: Basic Books Inc; 1983.
52. Bate P, Robert G. Experience-based design: from redesigning the system around the patient to co-designing services with the patient. Quality and Safety in Health Care. 2006;15(5):307-10.
53. Bate P, Robert G. Bringing user experience to healthcare improvement: The concepts, methods and practices of experience-based design: Radcliffe Publishing; 2007.
54. Thomson A, Rivas C, Giovannoni G. Multiple sclerosis outpatient future groups: improving the quality of participant interaction and ideation tools within service improvement activities. BMC Health Service Research. 2015;15(105).
55. Nicolini D. Practice Theory, Work, & Organization: An introduction. Oxford: Oxford University Press; 2012.
56. Nicolini D. Zooming in and zooming out: A package of method and theory to study work practice. In: Ybema S, Yanow D, Wels H, Kamsteeg F, editors. Organizational Ethnography. London, Thousand Oaks, New Delhi: Sage Publications; 2009. p. 120-38.
57. May C, Montori VM, Mair FS. We need minimally disruptive medicine. BMJ. 2009;339:b2803.
58. Mair F, May CR. Thinking about the burden of treatment. BMJ. 2014;349:g6680.

For peer review only - http://bmjopen.bmj.com/site/about/guidelines.xhtml

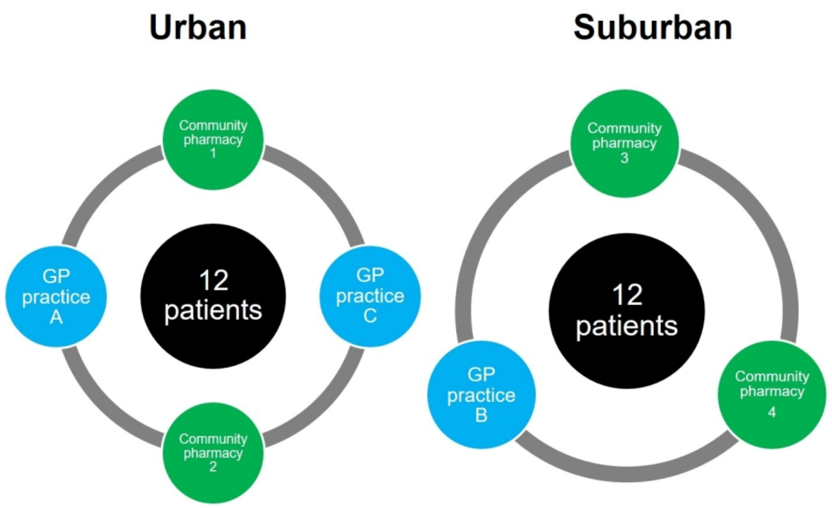

Figure 1. Study sites and research clusters

287x157mm (150 x 150 DPI)

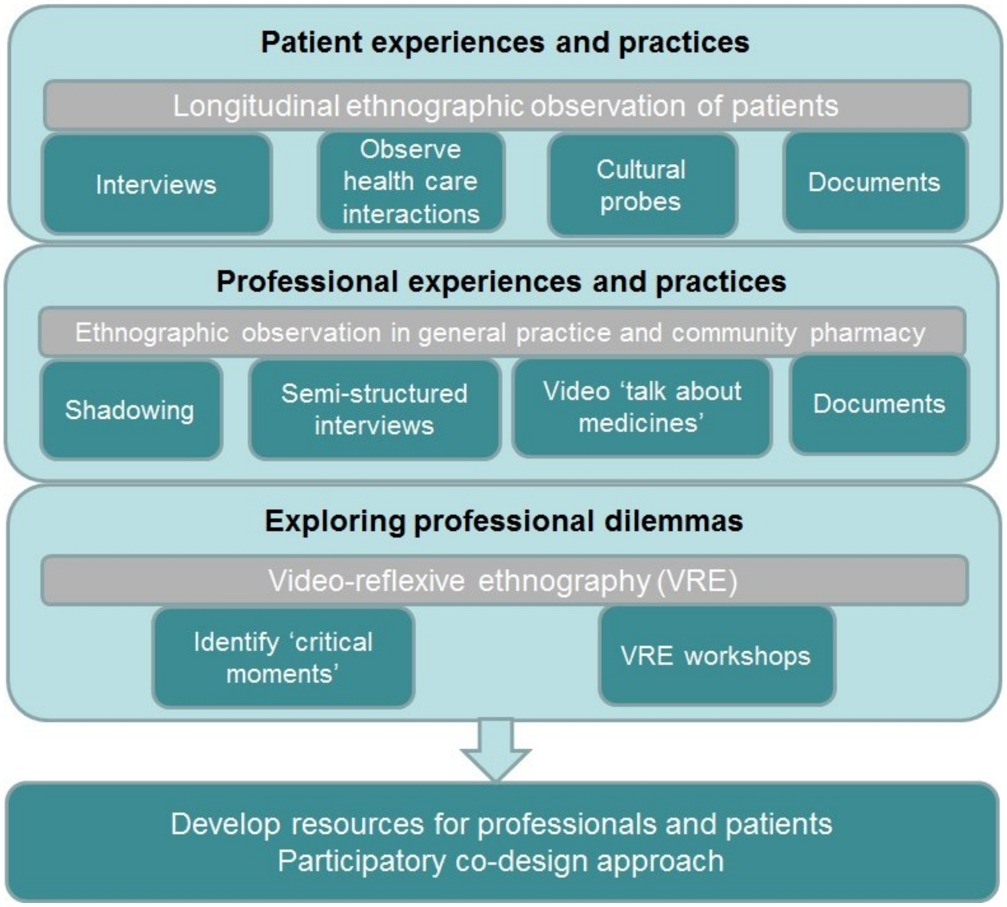

Figure 2: Methods and data sources

277x249mm (72 x 72 DPI)
